# Presence of *Plasmodium falciparum* strains with artemisinin-resistant K13 mutation C469Y in Busia County, Western Kenya

**DOI:** 10.1186/s41182-024-00640-1

**Published:** 2024-10-18

**Authors:** Mark Makau, Bernard N. Kanoi, Calvin Mgawe, Michael Maina, Mimie Bitshi, Edwin K. Too, Taeko K. Naruse, Hussein M. Abkallo, Harrison Waweru, Ferdinand Adung’o, Osamu Kaneko, Jesse Gitaka

**Affiliations:** 1 Centre for Malaria Elimination, Institute of Tropical Medicine, Mount Kenya University, Thika, Kenya; 2 Centre for Research in Infectious Diseases, Directorate of Research and Innovation, Mount Kenya University, Thika, Kenya; 3 Animal and Human Health Program, International Livestock Research Institute (ILRI), Nairobi, Kenya; 4 Department of Protozoology, Institute of Tropical Medicine (NEKKEN), Nagasaki University, Nagasaki, Japan; 5 Program for Nurturing Global Leaders in Tropical and Emerging Communicable Diseases, Graduate School of Biomedical Sciences, Nagasaki University, Nagasaki, Japan; 6 Centre for Infectious and Parasitic Diseases Control Research, Kenya Medical Research Institute, Busia, Kenya

## Abstract

Malaria remains a key health and economic problem, particularly in sub-Saharan Africa. The emergence of artemisinin drug resistance (ART-R) parasite strains poses a serious threat to the control and elimination of this scourge. This is because artemisinin-based combination therapies (ACTs) remain the first-line treatment in the majority of malaria-endemic regions in Sub-Saharan Africa. Certain single-nucleotide polymorphisms in the propeller domains of *Plasmodium falciparum* Kelch 13 protein (K13) have been associated with delayed parasite clearance in vivo and in vitro. These mutations serve as vital molecular markers for tracking the emergence and dispersion of resistance. Recently, there have been increasing reports of the emergence and spread of *P. falciparum* ART-R parasites in the Eastern Africa region. This necessitates continued surveillance to best inform mitigation efforts. This study investigated the presence of all reported mutations of K13 propeller domains in the parasite population in Busia County, Kenya, a known malaria-endemic region. Two hundred twenty-six participants with microscopically confirmed uncomplicated malaria were recruited for this study. They were treated with artemether–lumefantrine under observation for the first dose, and microscopic examination was repeated 1 day later after ensuring the participants had taken the second and third doses. *P. falciparum* DNA from all samples underwent targeted amplification of the K13 gene using a semi-nested PCR approach, followed by Sanger sequencing. The recently validated ART-R K13 mutation C469Y was identified in three samples. These three samples were among 63 samples with a low reduction in parasitemia on day 1, suggesting day 1 parasitemia reduction rate is a useful parameter to enrich the ART-R parasites for further analysis. Our findings highlight the need for continuous surveillance of ART-R in western Kenya and the region to determine the spread of ART-R and inform containment.

## Background

**Fig. 1:**
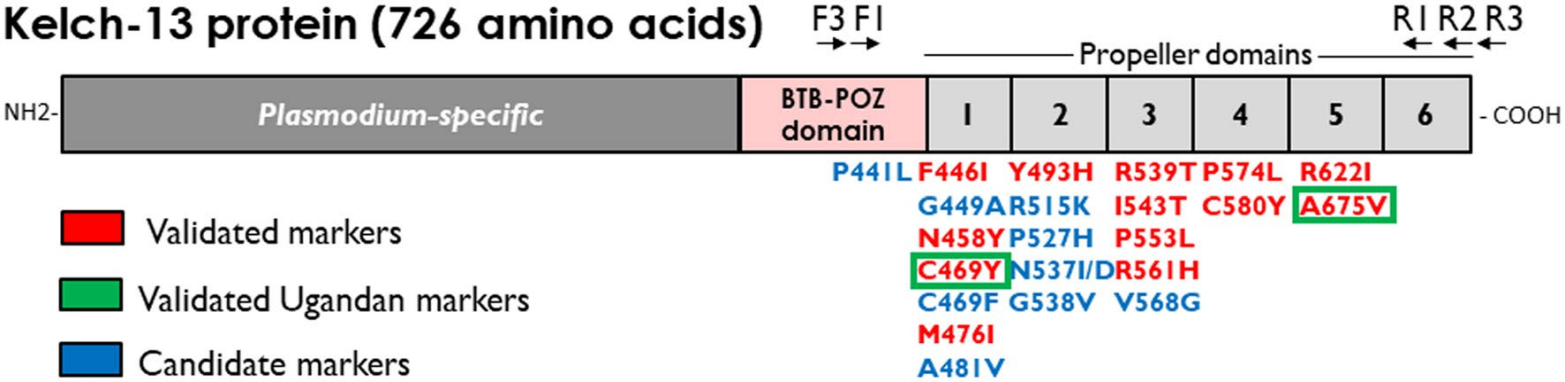
Schematic image of *P. falciparum* Kelch 13 protein with Plasmodium-specific sequence, a BTB–POZ domain, and 6 propeller domains. Locations of the oligonucleotide primers used in this study are indicated above the scheme (not to scale). Marker mutations validated (red) and candidate (blue) for artemisinin resistance [12] are shown below the scheme. Validated artemisinin-resistant mutations found in Uganda are enclosed within green boxes

Malaria remains a major public health challenge, claiming over 619,000 lives and causing more than 249 million clinical cases worldwide in 2022 alone, with most of the burden occurring in Sub-Saharan Africa [1]. Despite substantial efforts to reduce the incidence and mortality associated with malaria, progress has stalled in recent years [2]. This stagnation is particularly pronounced in East Africa, a region striving to control and ultimately eliminate malaria amidst myriad challenges [3, 4]. These include the emergence of drug-resistant malaria parasites, resistance of mosquito vectors to commonly used insecticides, genetic deletions undermining the utility of rapid diagnostic tests (RDTs), routine malaria commodity stock-outs, and limitations in the capacity for effective surveillance and response [5, 6]. The parasite *Plasmodium falciparum* accounts for most malarial infections in this region. The first cases of artemisinin resistance (ART-R) were reported in Southeast Asia in 2008 [7], and the *P. falciparum* Kelch 13 (K13) protein mutation, C580Y, was reported to be responsible for this ART-R in 2014 [7]. The C580Y mutation was spread over the years to different parts of Asia [8, 9], and now several mutations in the K13 protein in the BTB–POZ and Propeller domains have been validated as markers of delayed parasite clearance and they are defined as ART-R mutations, including F446I, N458Y, M476I, Y493H, R539T, I543T, P553L, R561H, and C580Y in Southeast Asia and C469Y, R515K, R561H, R622I, and A675V in Africa (Fig. 1) [10–12].

Studies in the Eastern Africa region have reported other K13 ART-R mutations independent from mutations reported in Asia; R561H in Rwanda [13], R561H, R622I and A675V in Tanzania [14, 15], and R561H and P441L in the Democratic Republic of the Congo [16], R622I in the Horn of Africa [17, 18], and A675V and C469Y in Uganda [19]. There is a strong expectation that these ART-R parasites will spread more due to the open border policies in the region, highly interconnected border communities, and lack of robust border malaria control strategies.

In Kenya, several novel and potential ART-R marker mutations, including R539T, N458T, R561H, N431S, and A675V, have been detected in the malaria-endemic areas of the country [20, 21]. However, most reported markers occur at low allelic frequencies. Recent studies in the coastal and Lake Victoria regions in Kenya reported no evidence of validated K13 resistance markers over a period of 13 years since the introduction of artemisinin-based combination therapies (ACTs) with artemether and lumefantrine (AL) as the first-line drugs for uncomplicated malaria in Kenya [22, 23].

Here, we make the first report of a K13 C469Y mutation in Kenya, a validated ART-R mutation originally reported from Uganda. This mutation has most likely spread from Uganda, where studies have shown increasing prevalence of this mutation over time [24]. Our findings elucidate the need for continuous surveillance of ART-R in this region and to establish systems for controlling the spread of resistance across border points of Kenya.

## Methods

### Study site

This study was conducted in Busia County, located on the Kenyan border with Uganda, and is characterised by moderate climatic conditions with average temperatures ranging between 20 to 28 ºC [25]. Malaria transmission is high and perennial, with seasonal peaks during long rain periods in May–June and short rain periods between October and November. The county is classified under lake endemic regions in Kenya, where the high temperature in these zones favours the malaria parasite sporogonic cycle, while the rainfall creates the breeding sites for malaria vectors. The population in this region is characterised by scattered homesteads. Despite being one of the smallest counties in Kenya, with a population of approximately 1.3 million people, it is also one of the biggest malaria-endemic areas with a prevalence of 39%. The situation is aggravated by factors such as limited access to healthcare facilities and poverty. In the 2020 Malaria Indicator Survey, malaria prevalence among children aged 6 months to 14 years in the Lake endemic region (where Busia is classified) was 42.4% by rapid diagnostic test (RDT) and 26.7% by microscopy. Notably, insecticide-treated net (ITN) coverage in 2020 was reported lower at 78% as compared to 2015, with 84% of households owning at least one ITN and 66% of persons reporting sleeping under a net the previous night in 2015 compared to 56% in 2020 [26].

### Selection of study participants

The study included participants who visited Busia County Hospital presenting with malaria symptoms. Informed consent was obtained from all participants, with participants < 18 years consent obtained from guardians. Patients with febrile clinical illnesses caused by pathogens other than malaria, pregnant, < 6 months, or those who had taken antimalarial medications 24 h before to visiting the health facilities were excluded from the study. *P. falciparum* infection was confirmed through microscopy, and only patients presenting with ≥ 1,000 parasites/μL were included. Patients who were able to have the blood taken on day 1 after the first AL administration were then selected for the analysis.

### Sample collection

Fifty microliters of blood samples were obtained from finger pricks using capillary tubes, and dried blood spots were prepared on the filter papers (Whatman^®^ 903 Proteinsaver cards; Cytiva, Marlborough, US) and appropriately marked with the participant’s unique study number. Patients received one oily donut and six doses of AL; the first AL dose was given under direct observation. A second set of samples was subsequently collected on day 1 following the initial administration of AL, after ensuring the participants had taken the second and third doses of AL. Patients were given the remaining AL tablets. Parasitemia was determined by counting the number of parasites per red blood cells on thin films. These parasitemias were used to calculate the parasite reduction rate.

### DNA extraction

DNA was extracted from all 226 samples using the protocol by Jaturas et al. [27], with a few modifications. A piece of paper 3 mm in diameter was punched from each dried blood spot, immersed in 1 mL of phosphate-buffered saline (PBS) for 30 min in a tube, and then the tube was centrifuged at 16,000 × g for 2 min. The supernatant was discarded, and then the papers were washed with PBS. Fifty microliters of nuclease-free water and 20 μL of 20% Chelex 100 were added to the tube and incubated at 99 ˚C for 10 min. Then, the supernatant was recovered after centrifugation at 16,000 × g for 1 min. DNA quantification was done by nanodrop spectrophotometry.

### *Amplification* of the *P. falciparum* K13 gene

A semi-nested approach was used to target the *P. falciparum* K13 gene. Primers were designed and their specificity was evaluated using PrimerBlast software (https://www.ncbi.nlm.nih.gov/tools/primer-blast) (Table 1 and Fig. 1). For the primary PCR (outer nested), 0.5 μL of KOD DNA polymerase (Sigma-Aldrich), 1 μL of 10 μM PfK13.F3 primer, 1 μL of 10 μM PfK13.R3 primer, 1 μL of DMSO, 1.5 μL of 25 mM MgSO_4_, 2.5 μL of KOD buffer ver2, 2.5 μL of 2 mM dNTPs, and 2 μL of the template DNA solution were mixed and topped up with nuclease-free water to a total reaction volume of 25 μL. The second run (inner nested) utilised primers PfK13.F3 and PfK13.R2 and 1 μL of the primary PCR product solution, with otherwise identical constituents as the first run. The optimised cycling conditions and expected fragment lengths are shown in Error! Reference source not found.. PCR products were subjected to 2% agarose gel electrophoresis and purified for sequencing.

**Table 1:** Nucleotide sequences of the oligonucleotide primers and optimized conditions for the amplification of *P. falciparum* K13 gene

Reaction	Primer name	Primer sequence 5'– > 3'	PCR product length	Cycling conditions
First round PCR (outer)	PfK13.F3	AGTGGAAGACATCATGTAACCAG	1143 bp	Denaturation 94˚C for 5 min, then 35 cycles of 94 ˚C for 30 s, 55 ˚C for 30 s, 68 ˚C for 1 min 30 s, and a final elongation of 68 ˚C for 5 min
	PfK13.R3	TGTGCATGAAAATAAATATTAAAGAAG		
Second round PCR (inner semi-nested)	PfK13.F3	AGTGGAAGACATCATGTAACCAG	1021 bp	
	PfK13.R2	CGGAGTGACCAAATCTGGG		
Sequencing primers	PfK13.F1 PfK13.R1	GCCTTGTTGAAAGAAGCAGAA GCCAAGCTGCCATTCATTTG		

### Sequencing

Sanger sequencing was performed using PfK13.F1 (5' GCCTTGTTGAAAGAAGCAGAA 3') and PfK13.R1 (5' GCCAAGCTGCCATTCATTTG 3') primers on the 3730xl DNA Analyzer (Thermo Fisher Scientific, Waltham, MA, USA) platform with a 2 × coverage for both forward and reverse reads. Chromas software Ver 2.6.6 (Technelysium Pty Ltd, Australia) was used for quality control. Multiple sequence alignment was then performed against the 3D7 K13 reference sequence (PF3D7_1343700) using MUSCLE software [28].

### Ethical considerations

Ethical approval for the research was obtained from the Mount Kenya University Institutional Scientific and Ethics Review Committee (MKUISERC) and the Research Ethics Committee, Institute of Tropical Medicine, Nagasaki University (Approval number 211216271).

## Results

### Participant characteristics

**Fig. 2:**
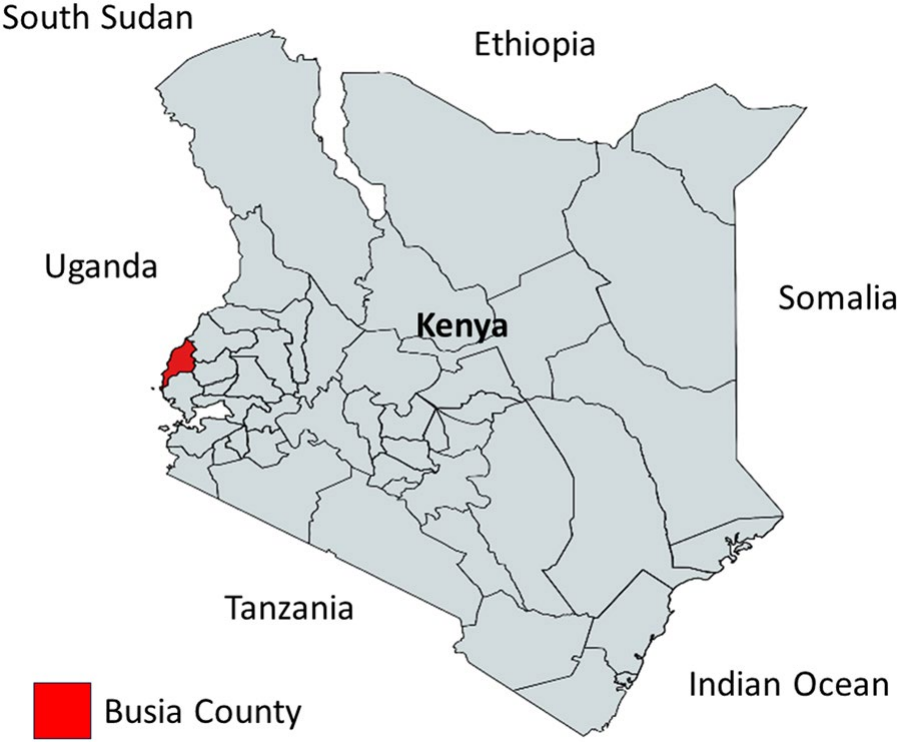
Geographical location of Busia County in Kenya. This is a malaria endemic region neighboring Uganda to the west and serves as a critical surveillance point based on cross-border interactions between citizens of both countries

**Fig. 3:**
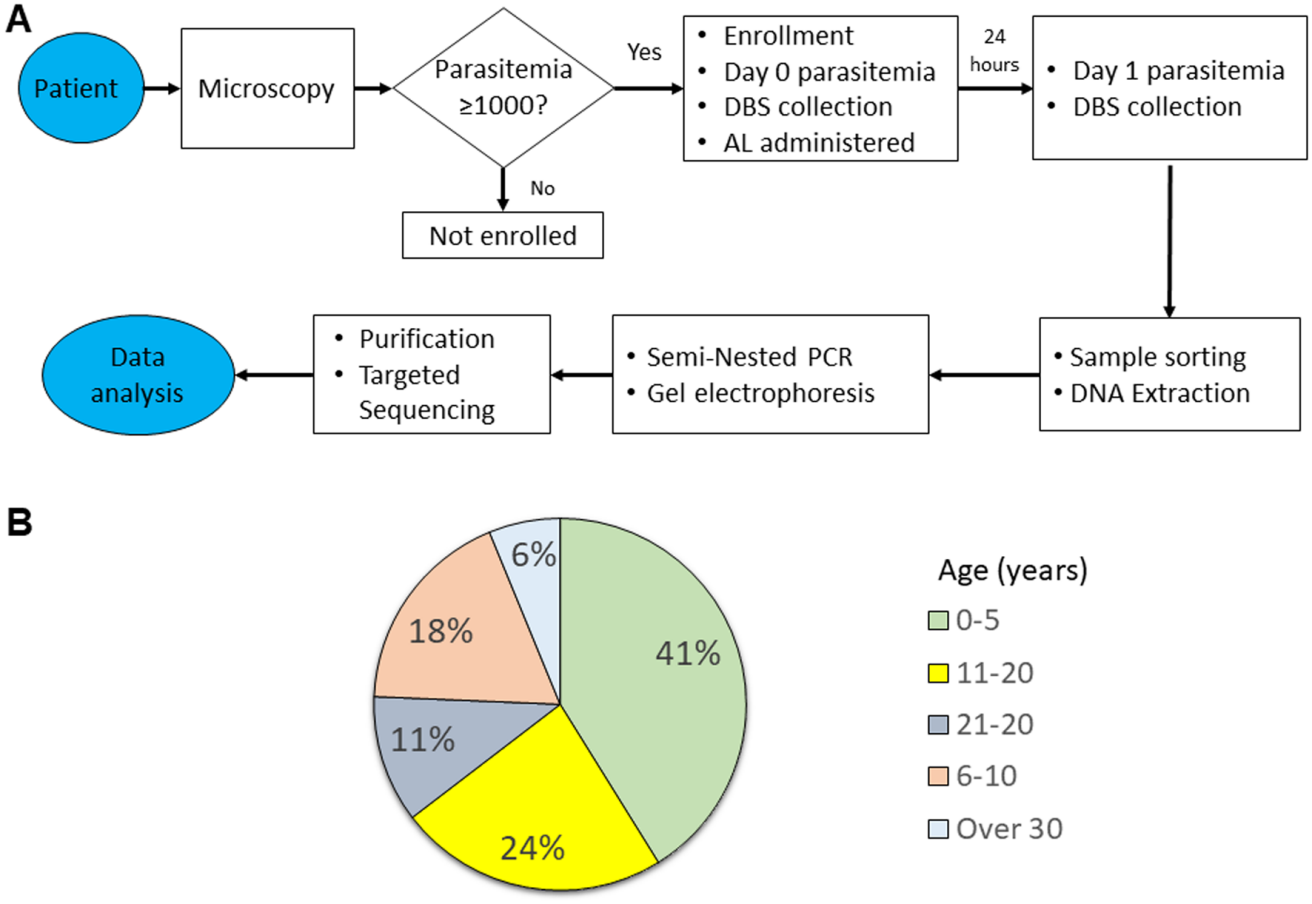
**A** Workflow used to select the study participants. AL, indicate artemether–lumefantrine; DBS, dried blood spot. **B** Study cohort stratified according to age groups. The majority of *P. falciparum* infections occurred in children under 5 years of age in this region

A total of 226 participants were recruited for this study from Alupe Level 4 Hospital, Busia County, western Kenya, from October to December 2022 (Fig. 2). These participants tested positive for *P. falciparum* and exhibited parasitemia levels above 1000 parasites/μL on day 0 baseline and therefore enrolled in the study (Fig. 3A**)**. Most participants were children below 5 years (41%), while those between 6 and 10 years represented 18% of those enrolled (Fig. 3B**)**. Participants older than 30 exhibited lower infection rates, likely due to naturally acquired immunity after prolonged exposure to *P. falciparum* (Fig. 3B). In this cohort, 63 individuals (27.9%) showed parasitemia on day 1 after AL administration that was equal to or more than 10% of the day 0 parasitemia.

### Analysis of the *P. falciparum* K13 gene

**Fig. 4:**
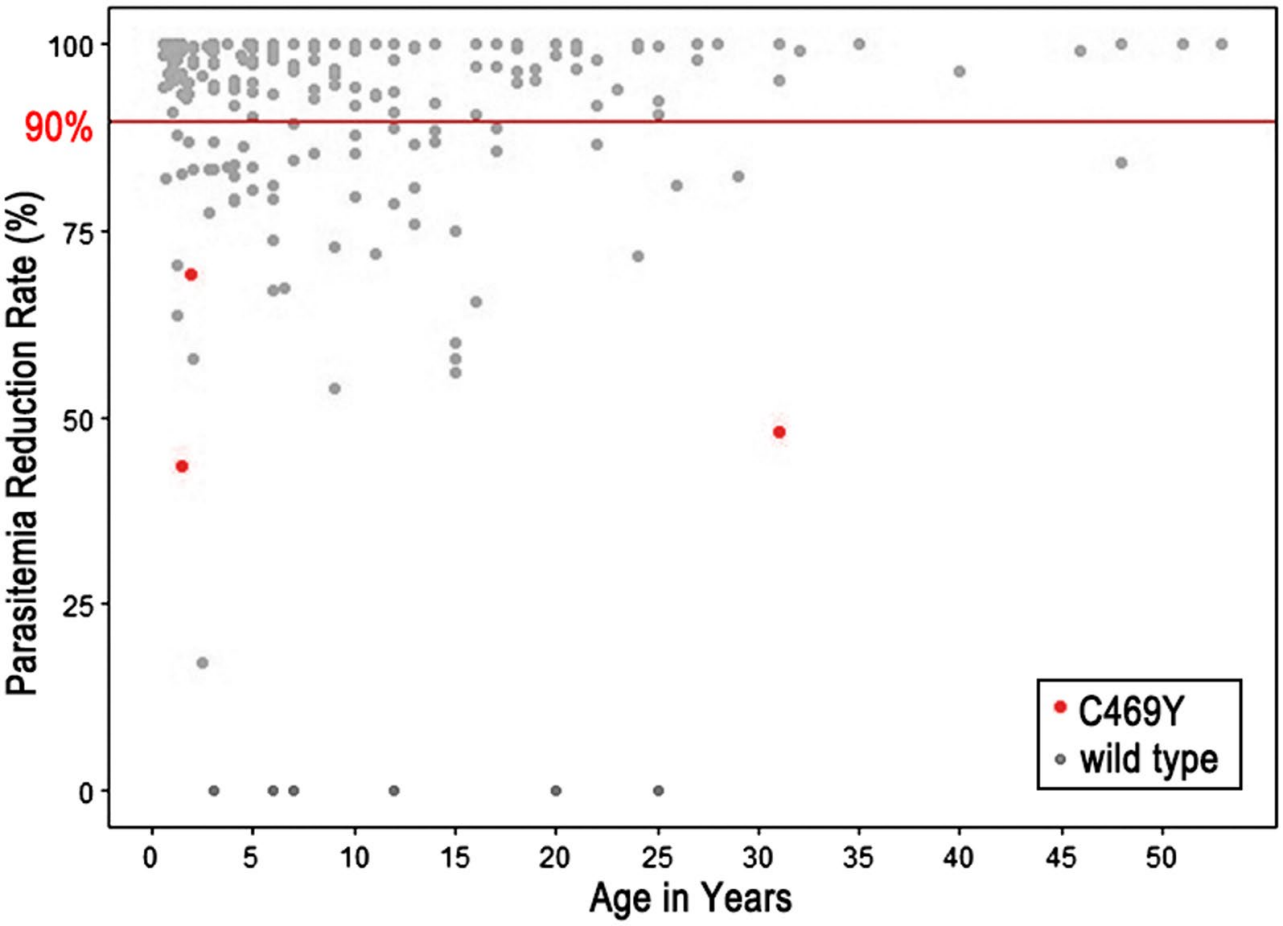
Parasitemia reduction rate per individual. The red line indicates the 90% parasitemia reduction. The red-labeled individuals present the K13 C469Y mutation. Sixty-three individuals exhibited parasitemia reduction rate equal to or less than 90% on day 1 after AL treatment

We sought to determine if any SNPs in the K13 gene that were associated with ART-R were present in 63 samples presenting day 1 parasitemia with equal to or more than 10% of the day 0 parasitemia. Analysis of multiple sequence alignment revealed a nonsynonymous mutation, a change of Cystine to Tyrosine at codon position 469 (C469Y) in 3 samples (Fig. 4). This mutation has been validated as a marker for ART-R in the Ugandan parasite population [19, 24]. The mutation occurred due to an SNP at nucleotide position 1,406 of the K13 gene, replacing codon TGC with TAC. This was 4.8% of the 63 samples presenting day 1 parasitemia with equal to or more than 10% of the day 0 parasitemia. Other nonsynonymous and synonymous mutations different from the reference wild-type sequence were not observed. Then, all remaining 163 samples, whose day 1 parasitemia was less than 10% of the day 0 parasitemia, were sequenced. All possessed wild-type K13 sequences except one which showed double peaks at nucleotide position 1,844 for C (wild type) and A, resulting P (wild type) and Q at amino acid position 615, respectively. K13 P615Q substituion has been reported in China–Myanmar border, but its contribution to ART-R is unknown [29]. The proportion of the C469Y mutation was therefore 1.3% of the total 226 samples recruited for this study. The ratio of the parasites possessing the K13 C469Y mutation was significantly higher in the group of samples presenting day 1 parasitemia with equal to or more than 10% of the day 0 parasitemia than the group of samples with the parasitemia on day 1 less than 10% of the day 0 parasitemia (p < 0.05 by two-tailed Fisher’s Exact test).

## Discussion

Ugandan-type K13 C469Y ART-R mutation was detected in 3 Kenyan *P. falciparum* patients in Busia County in 2022. Busia County is a malaria-endemic region bordering Uganda. Cross-border interactions between Kenyans and Ugandans could be a critical factor in the possible spread of *P. falciparum*-resistant mutants from Uganda into Kenya. There is a need to characterise the parasites further to determine their lineage, which will enhance our understanding of the evolution and spread of ART-R. This comprehensive approach will ensure more effective malaria control and prevention strategies.

AL is the first line of defence against symptomatic malaria in the East African region of Africa, including Kenya [1]. Despite the high efficacy presented by the drug studies, clinical cases of delayed parasite clearance have been reported over time, creating a need for continued therapeutic efficacy studies [30]. A rigorous investigation of clinical resistance to artemisinin should use one artemisinin-derivative only for treatment. However, to minimise the possibility of ART-R emergence, artemisinin-derivatives are currently administered with an efficacious partner antimalarial drug. We hypothesised that the effect of artemether on *P. falciparum* parasitemia 1 day after AL administration would be stronger than that of lumefantrine, based on the fact that the blood concentration of artemether increases rapidly. In contrast, that of lumefantrine, a slowly absorbed long-acting drug, increases slowly. While WHO protocols recommend day 3 samples for artemisinin resistance phenotyping, we examined the reduction rate of parasitemia on day 1 after drug administration to increase the likelihood of finding ART-R parasites. This is because previous reports indicated that more than 90% of parasites, on average, were cleared 24 h after treatment with artesunate [31, 32]. We found three samples with the ART-R type K13 sequence only in the group of samples showing a lower parasitemia reduction on day 1, which is significantly higher than the group of samples with parasitemia on day 1 less than 10% of the day 0 parasitemia. These findings suggest that examining the parasitemia reduction rate on day 1 may help identifying potential artemisinin-resistant parasites, as evidenced by the detection of the mutation in samples with lower parasitemia reduction. However, further investigation is required to establish if the proposed day 1 evaluation is indeed a useful strategy. We also do not consider that clinical resistance correlates with a decrease in parasitemia 1 day after drug administration, because the parasitemias of *P. falciparum* after antimalarial drug administration are affected by not only the killing effect of the drugs but also other factors, such as sequestration [31]. Nonetheless, our strategy is simple and practical when one wants to enrich ART-R parasites as much as possible, e.g., screening known resistant mutations, or identifying in vitro ART-R parasites. It is worth testing whether this strategy is effective in combination with other drugs used for ACTs.

ART partial resistance does not necessarily lead to treatment failure, especially if the partner drug is highly effective. However, the discovery of ART-R mutations in Rwanda [13], Tanzania [14, 15], and Uganda [19] was a warning sign of ART-R in the East African region. Based on the proximity of these countries to Kenya, the spread of these mutants into Kenya was imminent. From the molecular analysis of the K13 sequences, we confirm that the mutant C469Y, initially reported in Uganda, is now present in Kenya. The occurrence of this mutation in Kenya serves as a wake-up call for increased surveillance studies not only in the border region with Uganda but also with Tanzania and Rwanda.

## Conclusion

This study confirms the C469Y substituted mutation in the K13 gene in Kenyan *P. falciparum* isolates, with no Asian-type validated ART-R mutations in any of the samples. This finding underscores the importance of addressing the presence of this mutation in Kenya. Policymakers must take appropriate actions. Further molecular and phenotypic surveillance studies are essential in malaria-endemic regions of the country to inform policy-making and implementation, providing a comprehensive understanding of ART-R in Kenya. Therapeutic efficacy studies (TES) should also be conducted in Busia County to determine whether the current regimen is still efficacious against *P. falciparum* malaria. Additionally, implementing cross-border malaria surveillance is critical for monitoring and managing the spread of resistant strains between neighbouring countries. Finally, there is a need to characterise the parasites further to determine their lineage, which will enhance our understanding of the evolution and spread of ART-R in Busia. The strategy we have evaluated of prioritising patient blood samples with a lower parasitemia reduction on day 1 can speed up screening of known K13 mutations and establishing in vitro ART-R culture-adapted parasites from blood samples collected before treatment, which are valuable research samples for phenotypic analysis. This comprehensive approach will ensure more effective malaria control and prevention strategies.

## Data Availability

The datasets used and analysed during the current study are available from the corresponding author upon reasonable request.
